# Ode to positive constructive daydreaming

**DOI:** 10.3389/fpsyg.2013.00626

**Published:** 2013-09-23

**Authors:** Rebecca L. McMillan, Scott Barry Kaufman, Jerome L. Singer

**Affiliations:** ^1^Gifted Homeschoolers Forum Online, Chapel HillNC, USA; ^2^The Creativity Post, Chapel HillNC, USA; ^3^New York UniversityNew York, NY, USA; ^4^Yale UniversityNew Haven, CT, USA

**Keywords:** daydreaming, positive constructive daydreaming, volitional daydreaming, mind wandering, Jerome L. Singer, creativity, intelligence, default mode network

## Abstract

Nearly 60 years ago, Jerome L. Singer launched a groundbreaking research program into daydreaming (Singer, 1955, 1975, 2009) that presaged and laid the foundation for virtually every major strand of mind wandering research active today (Antrobus, 1999; Klinger, 1999, 2009). Here we review Singer’s enormous contribution to the field, which includes insights, methodologies, and tools still in use today, and trace his enduring legacy as revealed in the recent proliferation of mind wandering studies. We then turn to the central theme in Singer’s work, the adaptive nature of *positive constructive daydreaming*, which was a revolutionary idea when Singer began his work in the 1950s and remains underreported today. Last, we propose a new approach to answering the enduring question: Why does mind wandering persist and occupy so much of our time, as much as 50% of our waking time according to some estimates, if it is as costly as most studies suggest?

## INTRODUCTION

Over the past 60 years, researchers have assigned various names to the thoughts and images that arise when attention drifts away from external tasks and perceptual input toward a more private, internal stream of consciousness. The list includes daydreaming, thought intrusions, task irrelevant thoughts, spontaneous thought or cognition, stimulus independent thought, respondent thought, fantasy, task unrelated thought, task unrelated images and thought, internally generated thoughts, self-generated thought, absent-mindedness, zoning out, offline thought, undirected thought, unconscious thought, and mind wandering. This proliferation of terminology has obscured the common features of the phenomena under discussion and made it more difficult for current researchers to connect their work with the work of scholars who trod similar paths before them (Christoff, 2012).

Though mind wandering is the term most commonly used by cognitive researchers today, Singer’s preferred term is daydreaming. In his work, Singer differentiated between three styles of daydreaming: *positive constructive daydreaming*, characterized by playful, wishful imagery, and planful, creative thought; *guilty-dysphoric daydreaming*, characterized by obsessive, anguished fantasies; and *poor attentional control*, characterized by the inability to concentrate on either the ongoing thought or the external task (Singer, 1975). The three daydreaming styles Singer and colleagues identified are reflected in three major strands of mind wandering research active today: mind wandering as adaptive and beneficial, the relationship between mind wandering, especially rumination, and mood, and mind wandering as cognitive failure related to poor attentional control (Mooneyham and Schooler, 2013).

In this paper, we will confine ourselves to Singer’s original term, daydreaming, or the more commonly used term in recent studies, mind wandering, unless we are specifically referring to a more limited term used in a particular study. For the sake of clarity, we use Singer’s term, positive constructive daydreaming, when referring to the personally beneficial aspects of tuning into the internally generated content.

## BRIEF HISTORY

Singer’s investigations of daydreaming and imagination were already well established when his first doctoral student, John S. Antrobus, arrived at Teachers College, Columbia University in 1959 (Singer, 1975, 2009; Antrobus, 1999). By the time Singer and Antrobus published their first joint paper (Singer and Antrobus, 1963), Singer had already published eight papers directly discussing imagination, fantasy, and daydreaming (Singer, 1955, 1961; McCraven et al., 1956; Singer and Opler, 1956; Singer and McCraven, 1961, 1962; Singer and Schonbar, 1961; Singer and Rowe, 1962) and several more that touched on the same topics indirectly. Singer reported much of this early research, presenting a powerful argument for the adaptive role of daydreaming, in his seminal book “Daydreaming: An Introduction to the Experimental Study of Inner Experience” (Singer, 1966).

Over the next two decades, Singer and Antrobus enjoyed an enormously fruitful collaboration that produced key insights and developed or adapted to the study of thought flow tools and methodologies that are still in use today including *The imaginal processes inventory *(IPI; Singer and Antrobus, 1966; Antrobus et al., 1970) and *short imaginal processes inventory* (SIPI; Huba et al., 1981; Huba and Tanaka, 1983), *signal detection* (Antrobus et al., 1964; Singer, 1964a, 1975; Antrobus et al., 1966, 1967, 1970), an early precursor of *thought sampling* both in the laboratory (Singer and Antrobus, 1963) and out (Klos and Singer, 1981), which was retrospective rather than instantaneous as in later versions (Klinger et al., 1976; Csikszentmihalyi et al., 1977; Klinger, 1978; Hurlburt, 1979; Csikszentmihalyi and Graef, 1980; Prescott and Csikszentmihalyi, 1981; Csikszentmihalyi and Figurski, 1982); *ocular motility* (Antrobus et al., 1964; Singer and Antrobus, 1965; Singer et al., 1971), and *attention decoupling* (Antrobus et al., 1966, 1970; Singer, 1966; Antrobus, 1968).

At a time when most American psychologists associated fantasy and daydreaming with psychopathology, Singer and colleagues established that mind wandering is a normal, widespread, and adaptive human phenomenon that occupies a significant portion of waking thought (Singer and Antrobus, 1963; Singer, 1966). They also found that people are aware of daydreaming, can reliably report it on questionnaires (Antrobus et al., 1967) and that individuals differ in their daydreaming styles (Singer, 1974, 1975; Segal et al., 1980; Huba et al., 1981; Huba and Tanaka, 1983).

### INDIVIDUAL DIFFERENCES AND PERSONALITY

To illuminate individual differences in mind wandering tendencies, Singer and Antrobus developed the IPI (Singer and Antrobus, 1966, Revised 1970), a 344 item questionnaire probing such dimensions as daydream frequency, emotional content, sexual content, visual imagery, acceptance, and distractibility (Singer, 1975), followed by the SIPI (Huba et al., 1981; Huba and Tanaka, 1983). Factor analysis of the two inventories revealed three broad daydreaming styles: *positive constructive daydreaming*, *guilty-dysphoric* or *guilty-fear-of-failure daydreaming*, and *poor attentional control*. The validity of these three factors has been demonstrated repeatedly in the last 40 years across gender, ethnicity, life span, and mental health status (Giambra, 1974, 1977, 1979, 1980, 1989).

As early as 1960, Singer had already begun exploring the relationship between mind wandering and personality traits (Singer and Brown, 1960; Singer, 1961; Singer and McCraven, 1961, 1962; Singer and Schonbar, 1961). Decades later, working with Zhiyan, Singer found that the three styles of daydreaming identified in the factor analysis are associated with distinct Big Five Personality traits. Confirming an idea that Singer first explored in the early 1960s, Zhiyan and Singer found that positive constructive daydreaming was associated with Openness to Experience, reflecting curiosity, sensitivity, and exploration of ideas, feelings, and sensations. Poor Attentional Control was associated with low levels of Conscientiousness while Guilty-Dysphoric daydreaming correlated positively with neuroticism (Zhiyan and Singer, 1997).

## MODERN APPROACHES

Many of the methodologies and tools Singer and Antrobus pioneered in the 1960s and 1970s are still in active use today, either independently or in conjunction with brain imaging technologies. Most notably, Singer and Antrobus’ research foreshadowed Klinger’s proposal of a baseline state of mentation to which thought reverts in the absence of external demands (1971) and the discovery of the default mode network (DMN; Andreasen et al., 1995; Binder et al., 1999; Raichle et al., 2001) as well as its *partial* anti-correlation (Fox et al., 2005) with the executive attention network (EAN; Antrobus et al., 1966; Singer, 1966, 1974, 2009; Antrobus, 1968, 1999; Singer and Salovey, 1999; Kaufman and Singer, 2011). Although Singer and Antrobus lacked the brain imaging technology that facilitated the discovery of the DMN, they often remarked on the apparent competition between internally and externally produced information streams for limited attentional resources (Singer, 1966; Antrobus, 1968). However, they also recognized that the anti-correlation later described by Fox et al. (2005) is only partial because the human mind processes internal and external information streams both serially and, when conditions are right, as with repetitive or overlearned tasks or in familiar settings, in parallel (Singer, 1966, 1974, 2009; Antrobus, 1968; Antrobus et al., 1970).

### SIGNAL DETECTION AND THOUGHT SAMPLING

Numerous recent studies have also employed signal detection or thought sampling alone or in combination with brain imaging to elucidate the respective roles of the DMN and the EAN in mind wandering (Smallwood et al., 2005, 2007b, 2008a, 2012a; McKiernan et al., 2006; Kane et al., 2007; Mason et al., 2007; Christoff et al., 2009; McVay and Kane, 2009, 2010, 2012a,b; McVay et al., 2009; Andrews-Hanna et al., 2010; Killingsworth and Gilbert, 2010; Kam et al., 2011; Vanhaudenhuyse et al., 2011; Andrews-Hanna, 2012). Mason et al. (2007) combined three methods pioneered by Singer and Antrobus (thought sampling, ocular motility measurements and the IPI) in their study of the role of the DMN in mind wandering. After tabulating Voxel correlations with study participant’s scores on the daydream frequency scale of the IPI and a measure of eye movement frequency, they found that the incidence of mind wandering, the magnitude of BOLD signals observed in the DMN, and self-reported daydreaming frequency on the IPI were all positively correlated. Thus, some 40 years after it was first introduced, the IPI was used to corroborate early evidence showing a correlation between DMN activation patterns and mind wandering.

### EYE MOVEMENT

Another of Singer and Antrobus’ methods still in widespread use today is the measurement of eye movement in conjunction with mind wandering. This method is particularly common in studies examining the deleterious effects of mind wandering on reading (Schooler et al., 2004; Smallwood et al., 2008a,b; Reichle et al., 2010; Smilek et al., 2010). More recently, a number of studies have expanded beyond Singer and Antrobus’ ocular motility measurements to include measurements of pupil diameter (Einhäuser et al., 2008, 2010). In 2011, Smallwood et al. (2011a) found that pupil diameter serves as a reliable indicator of the decoupling of attention from perceptual input during offline thought.

### DECOUPLING

Over the last decade Smallwood, Schooler, and colleagues have invested significant energy investigating and refining the hypothesis first put forward by Singer and Antrobus (Antrobus et al., 1966; Singer, 1966; Antrobus, 1968) that mind wandering is associated with the decoupling of attention from perceptual input (Smallwood et al., 2003, 2008a, 2011a, 2012a,b). In the aggregate, these studies provide robust support for the decoupling hypothesis and Singer and Antrobus’ early observation that the incidence of mind wandering decreases as task demands or performance reward increase (Antrobus et al., 1966, 1970; Antrobus, 1968).

Most recently, Schooler et al. (2011) and Smallwood et al. (2012a) have elaborated the decoupling hypothesis further, suggesting that mind wandering consists of two core processes: perceptual decoupling and meta-awareness, the ability to take explicit note of one’s thoughts. In a study that examines the limits of perceptual decoupling, Kam et al. (2013) found that some attentional functions are maintained during mind wandering, most notably detection of unexpected changes in the environment.

### COGNITIVE CONTROL FAILURE

The vast majority of the research conducted over the last two decades portrays mind wandering as a cognitive control failure (McVay and Kane, 2010), highlighting its ill effects on reading comprehension, mood, memory, sustained attention, academic performance, IQ, and SAT test performance, and task-related processing (Teasdale et al., 1995; Smallwood et al., 2003, 2007a,b,c, 2008a,b, 2009a,b; Schooler et al., 2004; Kane et al., 2007; McVay and Kane, 2009, 2010, 2012a,b; McVay et al., 2009; Reichle et al., 2010; Smallwood and O’Connor, 2011; Mrazek et al., 2013). In a recent review of the costs and benefits of mind wandering, Mooneyham and Schooler (2013) identified 29 studies published since 1995 focused on the costs of mind wandering. On the benefits side of the ledger, they cited just six recent studies or publications noting the functional benefits of mind wandering.

Why such a gross imbalance in the recent literature? We know, not only from Singer’s work, but also from other studies that mind wandering is a universal human experience, affecting each of us countless times throughout the day (Klinger and Cox, 1987; Kane et al., 2007; Killingsworth and Gilbert, 2010). One large-scale study conducted via web application and mobile phones reported that, on average, mind wandering consumed 47% of the participants’ waking hours (Killingsworth and Gilbert, 2010). If the costs are so great and the benefits so scant, why do we spend so much of our time daydreaming? Why does mind wandering persist despite its costs? This question arises repeatedly throughout the literature (Klinger, 1971, 1999; Mooneyham and Schooler, 2013). Could it be that we are missing an important part of the story? While the costs of mind wandering are apparent and easily quantifiable, the benefits seem less obvious and tangible. They require us to dig a bit deeper.

## POSITIVE CONSTRUCTIVE DAYDREAMING

Singer and colleagues report many of the costs associated with mind wandering, yet the central theme of Singer’s large body of work is the manifestly positive, adaptive role that daydreaming plays in our daily lives (Singer, 1964b, 1966, 1974, 1975, 2009). We turn now to the benefits of daydreaming first described by Singer, then bolstered by recent studies exploring the adaptive role of the DMN and mind wandering on cognition.

Right from the start, Singer’s research produced evidence suggesting that daydreaming, imagination, and fantasy are essential elements of a healthy, satisfying mental life (Singer, 1966; Antrobus, 1999). His early research included studies looking at delayed gratification (Singer, 1955) and the interaction of imagination and waiting ability in young children (Singer, 1961). In another early study, Singer and Schonbar (1961) presented evidence of correlation between daydreaming frequency, measures of creativity, and storytelling activity. In the first paper co-authored with Antrobus, Singer explored the relationship between daydreaming, personality, divergent thought, creativity, planning, problem solving, associational fluency, curiosity, attention, and distractibility (Singer and Antrobus, 1963). Singer noted that daydreaming can reinforce and enhance social skills (Singer, 1964b), offer relief from boredom, provide opportunities for rehearsal and constructive planning, and provide a ongoing source of pleasure (Singer, 1966). In later work, Singer describes those who engage in positive constructive daydreaming as “happy daydreamers” who enjoy fantasy, vivid imagery, the use of daydreaming for future planning, and possess abundant interpersonal curiosity (Singer, 1974).

In a recent review, Schooler et al. (2011) suggested that positive constructive daydreaming serves four broad adaptive functions: *Future planning* which is increased by a period of self-reflection and attenuated by an unhappy mood; *creativity*, especially creative incubation and problem solving; *attentional cycling* which allows individuals to rotate through different information streams to advance personally meaningful and external goals; and *dishabituation* which enhances learning by providing short breaks from external tasks, thereby achieving distributed rather than massed practice. All four functions are present in Singer’s work, though his terminology differs.

### ADAPTIVE VALUE

In the last decade, studies investigating the role of the DMN in mind wandering bring the adaptive value of positive constructive daydreaming clearly into focus. Wang et al. (2009) provided evidence that the DMN activation and spontaneous thought are associated with offline memory consolidation. Baird et al. (2012) demonstrated that engaging in simple activities that permit daydreaming can promote creative incubation and problem solving.

Smallwood and colleagues undertook a series of studies exploring the temporal dimensions of mind wandering (Smallwood et al., 2009b, 2011b). Whereas previous research had established a link between negative mood and retrospective mind wandering (Smallwood et al., 2005; Smallwood and O’Connor, 2011), these studies reveal the adaptive benefits of prospective daydreaming. Smallwood et al. (2011b) found a strong link between self-reflection, autobiographical memory and future-oriented off task thought, suggesting all three cognitive processes are critical to our ability to simulate events in the future. This finding is highly consistent with Singer’s assertion that positive constructive daydreaming allows us to plan for and rehearse possible future scenarios (Singer, 1966).

In another study, Smallwood et al. (2013) investigated the effect of mind wandering on delay discounting finding that self-generated thought may contribute to the successful management of long term goals. Both the topic and the findings of this study echo some of Singer’s earliest work on the links between daydreaming, delayed gratification, and waiting behavior in young children (Singer, 1955, 1961; McCraven et al., 1956).

Baird et al. (2011) considered how positive constructive daydreaming contributes to the anticipation and planning of personally relevant future goals. They found that positive constructive daydreaming tends to be future-oriented and that those with greater working memory assets are more likely to engage in future-oriented daydreaming. Thus, idle working memory resources are essential to adaptive, prospective daydreaming. Whereas retrospective mind wandering tends to be loosely related to personal goals, Baird et al. (2011) conclude that spontaneous prospective thought is adaptive because it advances personally relevant goals. This research clearly echoes and meaningfully extends Singer’s work contrasting guilty-dysphoric daydreaming with positive constructive daydreaming.

In their noteworthy review, Immordino-Yang et al. (2012) highlight the value of “constructive internal reflection” for a wide range of socioemotional skills including compassion, moral reasoning, simulating the perspective of another person, understanding the implications of emotional responses, and deriving meaning from events and experiences. They argue that by imposing high attention demands on children in educational and other life contexts, we deprive them of the opportunity for the reflection that enables them to make personal meaning from their experiences and relationships. Once again, Singer’s work on the socioemotional aspects of positive constructive daydreaming reverberates in these findings.

While these studies focusing on the adaptive benefits of mind wandering may help to offset the dim view of mind wandering that persists in much of the literature, they reveal only part of the picture. We propose two areas of inquiry to expand our current understanding of mind wandering: (1) Positive Constructive Daydreaming can be volitional as well as unintentional and (2) Positive Constructive Daydreaming appears costly primarily when we measure it against external standards. Whereas the costs of daydreaming may be public and visible, the benefits are often private and hidden. To reveal the benefits of daydreaming, we must first identify an individual’s personally meaningful goals, aspirations and dreams and then consider how daydreaming supports or hinders realization of those goals.

## VOLITIONAL DAYDREAMING

Throughout the literature, mind wandering is portrayed as a mistake, a mental mishap, a cognitive failure. What is seldom acknowledged (with the exception of Christoff, 2012 and Smallwood, 2013) is that mind wandering can also be volitional. Individuals can *choose* to disengage from external tasks, decoupling attention, in order to pursue an internal stream of thought that they expect to pay off in some way. The pay off may be immediate, coming in the form of pleasing reverie, insight, or new synthesis of material, or it may be more distant as in rehearsing upcoming scenarios or projecting oneself forward in time to a desired outcome. Projection backward in time to reinterpret past experiences in light of new information is also a possibility (Smallwood et al., 2009b, 2011b). All of these activities, which take place internally, sheltered from the demands of external tasks and perception, offer the possibility of enormous personal reward. These mental activities are, in fact, central to the task of meaning making, of developing and maintaining an understanding of oneself in the world (Immordino-Yang et al., 2012).

Singer hinted at the possibility of volitional daydreaming saying, “Our human condition is such that we are forever in the situation of deciding how much attention to give to self-generated thought and how much to information from the external social or physical environment” (Singer, 1993). Recent research revealing the involvement of the lateral prefrontal cortex in mind wandering also suggests the possibility of volitional mind wandering (Teasdale et al., 1995; Gilbert et al., 2005; Christoff et al., 2009; Spreng et al., 2010; Smallwood et al., 2012a).

Despite these hints, previous research tends to emphasize the unintentional onset of mind wandering, suggesting that it is automatically triggered by cues that may fall within or outside our conscious awareness (Klinger, 1971, 2009; Bonanno and Singer, 1993; Giambra, 1995; McVay and Kane, 2010). The suggestion seems to be that our minds wander against our best wishes; that our drifting mind is beyond our control. Certainly a large share of mind wandering occurs without permission or awareness. But some mind wandering occurs because we actively *choose* to decouple from external tasks and perceptions and focus instead on an internal stream of thought with full awareness both of the choice being made and the contents of consciousness.

While everyone may be capable of such volitional daydreaming, the capacity to switch at will between inner and outer streams of consciousness may be more fully developed in some than in others. Extrapolating from Singer’s work with both Antrobus and Zhiyan, we can easily imagine that this may be an area in which individual differences come into play. It stands to reason that positive constructive daydreamers, those who are most open to experience and who consider daydreaming a positive experience, would be most likely to engage in volitional daydreaming. On the other hand, those who experience mind wandering as less or unpleasant would be less likely to engage in volitional mind wandering. This latter group would include ruminators and guilty-dysphoric daydreamers whom Singer and Zhiyan found to show high levels of neuroticism and poor attentional control daydreamers who tend to show low levels of conscientiousness and have a hard time focusing on the internal information stream or external task demands (Zhiyan and Singer, 1997).

It seems likely that the ability to engage in volitional daydreaming, i.e., to switch easily back and forth between different streams of consciousness, might be sensitive to practice effects. Choosing to disengage from external tasks, decouple, turn attention inward, and follow an internal stream of thought with full awareness undoubtedly requires skill. The process can break down in a number of places along the way: at the decision point, decoupling, the switch from outer to inner streams of consciousness, or meta-awareness. But the more a person does it, the easier it is likely to become.

Mind wandering, which occurs as a happy or unhappy accident for some, is consciously cultivated by others. Surprisingly, it appears that no one has yet attempted to correlate frequency of daydreaming with Singer’s three daydreaming styles. Research has shown, however, that those who report more daydreaming do, in fact, daydream more often (Antrobus et al., 1967). Hence, frequent daydreamers, especially those who report positive constructive daydreaming, would be an obvious place to start the search for volitional daydreaming.

What neural architecture supports volitional daydreaming? Most likely, volitional daydreaming involves the interaction of multiple large-scale brain networks (Bressler and Menon, 2010). For instance, Smallwood et al. (2012a) argue that the ability to generate and sustain an internal train of thought is supported by cooperation between the EAN and the DMN (also see Christoff et al., 2009). Another important player is most certainly the salience network (SN), which includes the anterior cingulate cortex, presupplementary motor area, and anterior insulae. The SN is important for the dynamic and flexible switching between the EAN and the DMN (Sridharan et al., 2008; Bressler and Menon, 2010; Bonnelle et al., 2012). Since the SN plays such a crucial role in signaling the need to change streams of consciousness, an inability to activate the SN might not only lead to difficulty with cognitive control (e.g., Bonnelle et al., 2012), but may also limit the ability to access a positive and constructive inner stream of consciousness on demand (Bonnelle et al., 2012).

Whatever the neural mechanisms involved, a deeper understanding of volitional positive constructive daydreaming can add to our understanding of why mind wandering is so pervasive. But there is more work to be done. It’s time to take a closer look at how we calculate the costs and benefits of mind wandering.

## PERSONAL GOALS

As noted above, most recent studies depict mind wandering as a costly cognitive failure with relatively few benefits (Mooneyham and Schooler, 2013). This perspective makes sense when mind wandering is observed by a third party and when costs are measured against externally imposed standards such as speed or accuracy of processing, reading fluency or comprehension, sustained attention, and other external metrics.

There is, however, another way of looking at mind wandering, a personal perspective, if you will. For the individual, mind wandering offers the possibility of very real, personal reward, some immediate, some more distant. These reward include self-awareness, creative incubation, improvisation and evaluation, memory consolidation, autobiographical planning, goal driven thought, future planning, retrieval of deeply personal memories, reflective consideration of the meaning of events and experiences, simulating the perspective of another person, evaluating the implications of self and others’ emotional reactions, moral reasoning, and reflective compassion (Singer and Schonbar, 1961; Singer, 1964b; Singer, 1966, 1974, 1975, 1993, 2009; Wang et al., 2009; Baars, 2010; Baird et al., 2011, 2012; Kaufman and Singer, 2011; Stawarczyk et al., 2011; Immordino-Yang et al., 2012; Kaufman, 2013). From this personal perspective, it is much easier to understand why people are drawn to mind wandering and willing to invest nearly 50% of their waking hours engaged in it.

We mind wander, by choice or accident, because it produces tangible reward when measured against goals and aspirations that are personally meaningful. Having to reread a line of text three times because our attention has drifted away matters very little if that attention shift has allowed us to access a key insight, a precious memory or make sense of a troubling event. Pausing to reflect in the middle of telling a story is inconsequential if that pause allows us to retrieve a distant memory that makes the story more evocative and compelling. Losing a couple of minutes because we drove past our off ramp, is a minor inconvenience if the attention lapse allowed us finally to understand why the boss was so upset by something we said in last week’s meeting. Arriving home from the store without the eggs that necessitated the trip is a mere annoyance when weighed against coming to a decision to ask for a raise, leave a job, or go back to school.

Some recent studies (Baird et al., 2011, 2012; Smallwood et al., 2011b; Immordino-Yang et al., 2012) have provided glimpses of how mind wandering or “constructive, internal reflection” (Immordino-Yang et al., 2012) might benefit the individual, but we are just beginning to scratch the surface. To gain a fuller understanding of the benefits of positive constructive daydreaming we need to apply tools and metrics (as in Klinger et al., 1980; Hoelscher et al., 1981; Nikles et al., 1998; Cox and Klinger, 2011; Klinger and Cox, 2011) that enable us identify the personally meaningful goals, aspirations, and dreams of individuals and determine how mind wandering supports or undermines those goals. Given the highly personal nature of mind wandering, we need a new focus and new metrics.

### INTELLIGENCE

Intelligence theories provide an interesting parallel. Traditional theories of intelligence emphasize cognitive control, deliberate planning, and decontextualized problem solving as the essence of human intelligence (Kaufman, 2011). This is largely due to the purpose of the first intelligence test: to identify students in need of alternative education. Because intelligence tests were designed to predict school grades, the tests were intentionally designed to measure the ability to profit from explicit instruction, concentrate on an external goal, and engage in abstract reasoning. Therefore it should come as no surprise that IQ test performance is strongly associated with activation of the EAN (e.g., Jung and Haier, 2007; Barbey et al., 2012).

While the cognitive functions measured on traditional metrics of intelligence are undoubtedly important contributors to intellectual functioning, they are mostly decontextualized. Rarely are the test takers allowed to dip into their inner stream of consciousness and produce an original response that incorporates self-relevant information. To help correct this imbalance in the literature, Kaufman (2013) proposed the Developmental Theory of Personal Intelligence. According to the theory, intelligence is the dynamic interplay of engagement and ability over an extended period of time in pursuit of personal goals (Kaufman, 2013). The emphasis is adaptation to task demands that are relevant to attaining one’s personal goals, not just adaptation to the external goals dictated by educators and experimental psychologists. Therefore, the theory takes into account an individual’s personal goals, and considers both controlled forms of cognition (e.g., working memory, attentional focus, etc.) *and* spontaneous forms of cognition (e.g., intuition, affect, insight, implicit learning, latent inhibition, and the spontaneous triggering of episodic memories and declarative knowledge) are important potential contributors to that personal adaptation.

This broadened conceptualization of human intelligence is in line with the plethora of research we already reviewed in this paper on the adaptive value of positive constructive daydreaming. Research shows that when daydreaming, the contents of consciousness tend to be focused on upcoming personally meaningful events, indicating that they may play a role in autobiographical planning (Smallwood et al., 2009b; Morsella et al., 2010). In particular, Klinger (1999) showed that people’s daydreams and night dreams reflect “current concerns” ranging from constant thought of incomplete tasks to unresolved desires, ranging from sexual and social strivings to altruistic or revenge urges and the panoply of human motivations.

This deeply personal conceptualization of intelligence is also in line with the latest research in cognitive neuroscience. D’Argembeau et al. (2010) found that imagining personal future events elicited stronger activation in two key hubs of the DMN – the ventral medial prefrontal cortex (MPFC) and the posterior cingulate cortex (PCC) – compared to imagining non-personal future events. The researchers suggest that these brain areas support a collection of mental processes that evaluate, code, and contextualize the relevance of mental representations with regard to personal goals. Since traditional measures of intelligence do not allow individuals to imagine personal future events, or connect the test information to their large storehouse of episodic memories, functioning of these key regions of the DMN are ignored in the assessment of intellectual functioning.

Another key implication is that sometimes behavior that appears “unintelligent” measured by external standards may actually be quite intelligent as judged by its relevance to achieving personally meaningful goals. Importantly, these different ways of being “smart” can conflict with each other. According to the neural global workspace theory of consciousness, different streams of consciousness compete for access to a global conscious workspace (Baars, 1993). This may explain why the EAN and the DMN tend to be anticorrelated (Fox et al., 2005). Daily life often demands that we choose one information stream or the other. For instance, in a decontextualized educational context, or in a cognitive psychology experiment, the ability to concentrate on a task requires silencing the inner chatter. Vice versa, when we would like to dip into our inner stream of consciousness, we must block out our external percepts (Dehaene and Changeux, 2005; Smallwood et al., 2011b; Kam et al., 2013).

However, as Kam et al. (2013) point out, when the EAN works in concert with the DMN to sustain an inner train of thought, selective attention processes are not absent – they just are turned inward to select the most relevant associations and ideas that emerge from episodic memory. This has important implications, because traditional views of selective attention erroneously assume that the main function of the EAN is to select relevant stimuli from the *external environment* for deliberate, conscious processing. However, these traditional models miss a key feature of human cognition: when working in cooperation with the DMN, the EAN is equally equipped to select relevant episodic associations that can help keep an inner stream of thought both positive and constructive.

## CONCLUSION

Whatever aspect of mind wandering current researchers might wish to pursue, it is likely that Singer considered the question first and made as thorough an investigation as the technology of the day would allow. His research serves as a solid foundation and springboard for all who come after him and share his fascination with positive constructive daydreaming, mind wandering, and the imaginative capabilities of the human mind. Our field owes him an enormous debt of gratitude and respect. We are happy to do our small part to honor that debt.

In closing, we should note that much of what we have presented here first emerged not from an intense period of methodical, laser-like focus but from periods of diffuse inward focus in which our minds were not merely permitted but *willed* to roam freely within our respective mental landscapes. Thanks to nearly six decades of Singer’s work, we were confident that was where our best and most productive insights would be found.

## Conflict of Interest Statement

The authors declare that the research was conducted in the absence of any commercial or financial relationships that could be construed as a potential conflict of interest.
